# Neutrophil store-operated Ca^2+^ entry: A correctable biomarker of cystic fibrosis lung disease progression

**DOI:** 10.1016/j.jcf.2025.08.014

**Published:** 2025-08-27

**Authors:** Joe A Wrennall, Matthew GS Biggart, Charles D Bengtson, M Flori Sassano, Robert Tarran

**Affiliations:** a Department of Cell Biology and Physiology, University of North Carolina at Chapel Hill, NC, 27599; b Division of Genetic, Environmental and Inhalational Disease, Department of Internal Medicine, Kansas University Medical Center, Kansas City, KS, 66103, USA; c Division of Pulmonary, Critical Care and Sleep Medicine, Department of Internal Medicine, Kansas University Medical Center, Kansas City, KS, 66103, USA

**Keywords:** Orai1, Icrac, CFTR, neutrophil elastase

## Abstract

**Rationale:**

People with cystic fibrosis (pwCF) exhibit chronic and hyperactive neutrophilia which results in a progressive loss of lung function. CF neutrophils have elevated store operated Ca^2+^ entry (SOCE) relative to healthy non-CF neutrophils, which contributes to persistent neutrophilia. The vast majority of pwCF now take CFTR modulators such as elexacaftor/tezacaftor/ivacaftor (ETI), which effectively increase CFTR function in multiple organs including the lung. However, ETI’s impact on neutrophils is poorly understood. Orai1 is a plasma membrane Ca^2+^ channel that contributes to SOCE. We have developed a novel peptide (ELD607) that specifically inhibits Orai1, which we evaluated in CF neutrophils.

**Objectives:**

To characterize Orai1/SOCE in neutrophils from pwCF taking ETI, and to evaluate the impact of SOCE inhibition by ELD607 on pwCF neutrophil Ca^2+^ signaling/function.

**Methods:**

Peripheral blood neutrophils were isolated by negative selection. SOCE was characterized using fluorescent approaches. Protein expression was characterized by proteomics and confocal microscopy. Neutrophil degranulation was measured using a multiplex assay.

**Measurements and main results:**

Proteomic analysis revealed major global differences between non-CF and pwCF neutrophils, despite use of ETI. Several proteins involved in SOCE, including Orai1, were significantly elevated in pwCF neutrophils. ELD607 dose-dependently inhibited SOCE, leading to reduced neutrophil degranulation. Ca^2+^ homeostasis was significantly elevated in pwCF compared to non-CF neutrophils. ELD607-sensitive SOCE inversely correlated with lung function (FEV1pp).

**Conclusions:**

Our findings highlight SOCE as a novel biomarker of CF lung disease. ELD607 can be used to reduce SOCE and subsequent degranulation in CF neutrophils. We therefore hypothesize that ELD607 may be of benefit in the management of inflammation in pwCF.

## 1. Introduction

Cystic fibrosis (CF) is an autosomal recessive disease caused by mutations in the cystic fibrosis transmembrane conductance regulator (CFTR) anion channel. In the lung, loss of CFTR function impairs ion and water homeostasis leading to airway dehydration, impaired mucociliary transport and chronic airway infections [1]. Subsequent cycles of infection and neutrophilic inflammation lead to a progressive loss of lung function [2–4]. Indeed, excessive and sustained neutrophilic inflammation is a hallmark of CF lung disease [1,5]. Recently, highly effective CFTR modulators such as ETI have become clinically available [4]. These therapies utilize a combination of small molecules that improve CFTR function leading to improved clinical outcomes including reduced sweat chloride levels and increased forced expiratory volume in 1 second (FEV1). The long-term benefits of ETI are still being assessed, but, data suggest that lung function is not fully normalized [6] and bacterial infections are not resolved [4,7]. Further, single cell RNAseq demonstrated that ETI only partially restored inflammatory gene networks in CF immune cells (neutrophils and macrophages) [8]. Similarly, CF inflammation appeared to be reduced but not fully normalized by ETI [9].

Neutrophils are early responders of the innate immune system, which act to neutralize pathogens through the release of antimicrobial granule contents including cytokines, proteases and reactive oxygen species [10]. However, if not rapidly resolved, chronic neutrophilia is well known to damage host tissues [2–4]. CF neutrophils display functional defects that promote poorly-resolving inflammatory responses and excessive degranulation [11–14]. Moreover, CF peripheral blood neutrophils exhibited impaired bacterial clearance [15]. Neutrophil activation is initiated by the recognition of inflammatory stimuli by cell surface G protein-coupled receptors (GPCRs). Activation of these GPCRs triggers the emptying of endoplasmic reticulum (ER) Ca^2+^ stores. Depletion of the ER Ca^2+^ stores is detected by stromal interacting molecule 1 (STIM1), which relocates to ER/plasma membrane junctions where it activates plasma membrane Ca^2+^ channels such as Orai1 [16], initiating Ca^2+^ influx. Collectively, this process is called store operated Ca^2+^ entry (SOCE) and it promotes altered transcriptional activity, neutrophil activation, chemotaxis, and/or degranulation [17]. CF neutrophils have impaired Ca^2+^ homeostasis which may underlie their hyper-inflammatory phenotype [12]. Similarly, we have previously found that, in the absence of CFTR modulators, Orai1 activity is increased in CF patient lungs and blood neutrophils, suggesting that increased SOCE may underlie excessive neutrophilia in the CF lung [18].

Bacterial killing by CF neutrophils improved following *ex vivo* treatment with the CFTR potentiator Kalydeco [12]. For this reason, a re-evaluation of CF neutrophil Ca^2+^ homeostasis/SOCE in the age of CFTR correctors/modulators is warranted. We have previously identified the 6^th^ alpha helix (α6) of short palate lung and nasal epithelial clone 1 (SPLUNC1) as an endogenous inhibitor of Orai1 and SOCE [19]. Thus, we hypothesized that α6 peptidomimetics could be used as novel immunomodulators for the treatment of hyperactive Ca^2+^ signaling in CF neutrophils. Since SPLUNC1 is rapidly degraded by neutrophil elastase [20], we developed ELD607, a size-optimized SPLUNC1/α6 peptidomimetic that is protease-resistant and stable in CF patient-derived neutrophil elastase [21]. In murine models of bacterial lung infections, ELD607 reduced neutrophilia and lung damage leading to increased survival [21]. Accordingly, we characterized SOCE/Orai1 in non-CF and CF peripheral blood neutrophils and evaluated the impact of ELD607 treatment on SOCE and neutrophil degranulation.

## 2. Methods

Full methods are available in the online supplement.

### 2.1. Participant enrollment

Adults with CF at UNC-Chapel Hill or KUMC Adult CF care center outpatient clinics were eligible for enrollment. Clinical information, including most recent spirometry values, were abstracted from the medical record. Venipuncture was performed to obtain peripheral blood for neutrophil isolation. The study was approved by the local institutional review boards.

### 2.2. Neutrophil isolation

Neutrophils were immediately isolated by negative selection from deidentified blood samples using an EasySep human neutrophil isolation kit (Stem Cell Technologies) as described [22]. Cytosolic Ca^2+^ was measured using Fluo-4 as described [23]. Protein lysate from isolated blood neutrophils was characterized by liquid chromatography with tandem mass spectrometry (LC-MS/MS) as described [24]. Degranulation products in media were quantified by Luminex assay using a custom Bio-Plex human kit (BioRad) per the manufacturer’s protocol to ≥ 99% purity. Detailed methods for all experiments and measurements are provided in an online data supplement.

### 2.3. Proteomics

Protein lysate from isolated blood neutrophils was subjected to liquid chromatography with tandem mass spectrometry (LC-MS/MS) as described [24].

### 2.4. Fluo-4 Ca^2+^ assays

Isolated neutrophils were incubated with peptide or vehicle control for 3 h. For the final hour of treatment, cells were loaded with Fluo-4 direct (Thermofisher) for 45 min at 37°C and 15 min at room temperature as described [25].

### 2.5. Immunocytochemistry

Isolated neutrophils were treated for 3 h with vehicle, 10 μM ELD607 or scrambled ELD607 control and were fixed in 4% PFA, washed in PBS, permeabilized in 0.1% Triton X-100 in PBS and blocked overnight (0.1% Triton X-100, 10% FBS, 5% NGS in PBS). Cells were incubated in 1:500 rabbit anti-Orai1 primary antibody (Sigma, SAB3500412) overnight at 4°C, washed 3 times in PBS and stained for 3 h at 4°C in 1:2000 goat anti-rabbit IgG DyLight-633 secondary antibody (ThermoScientific, 35562). Cells were mounted in VECTASHIELD PLUS mounting media with DAPI and imaged on a Leica SP8 confocal microscope (DyLight 633: excitation 594 nm, emission 600–764 nm; DAPI excitation 405 nm, emission 410–480 nm) as described [25].

### 2.6. Quantification and statistical analysis

Proteins were identified and quantified from LC-MS/MS data with Spectronaut v17 using a Uniprot Human database. Proteins where only one peptide was identified were excluded from the analysis, as were keratin-type proteins (supplemental table 1). Further data analysis (t-tests, visualization) were performed in Spectronaut. Data were normalized, and outliers were identified and removed. Heat maps were rendered using Morpheus (https://software.broadinstitute.org/morpheus). All other statistical analysis was performed using GraphPad Prism 9.0. Data were analyzed using Student’s t-test or ANOVA followed by Sidak’s post-test. Paired data were analyzed using a mixed effects model with Holm-Sidak post-test. Dose response curves were compared using exact sum of squares f test.

## 3. Results

### 3.1. CF neutrophils have an altered cellular proteome

**Fig. 1.**
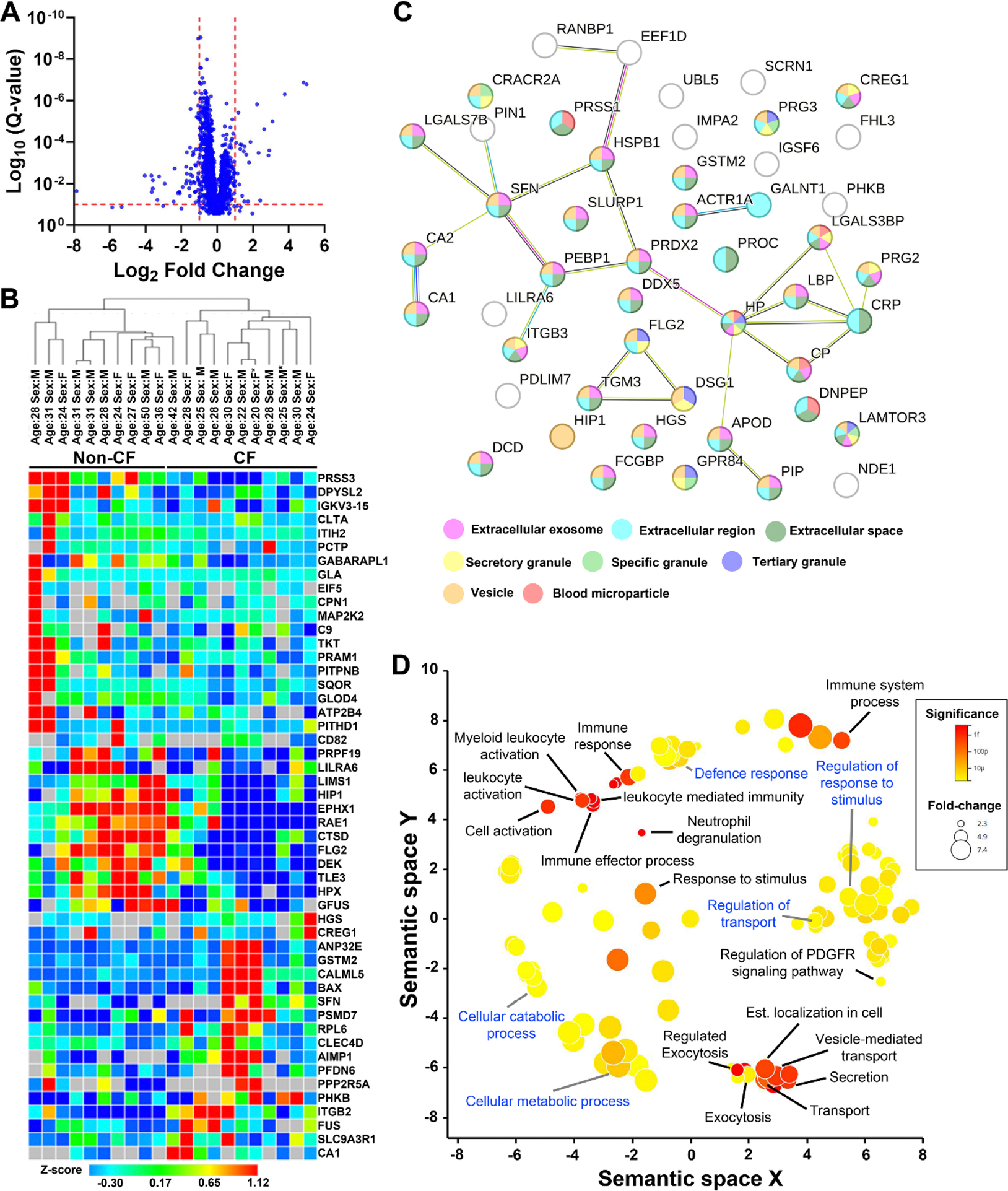
CF peripheral blood neutrophils have an altered proteome. (A) Mean relative abundance of detected proteins in CF neutrophils (n = 11 donors) compared to non-CF neutrophils (n = 10 donors). Red dashed lines indicate q=0.1 and ± 2-fold change. (B) Heat map showing the relative abundance (Z-score) of proteins with 2-fold or more change in relative abundance. Donors have been arranged based on hierarchical clustering (one minus Pearson method, network lines above heat-map). Coloration indicates Z-score. Grey boxes indicate undetected proteins. (C) Ontology network of significantly altered proteins with ≥2-fold change in mean abundance. Nodes are color coded to indicate association to cellular compartments. Lines denote known interactions between proteins. (D) Changes in biological processes identified by ontology organized within a semantic map. Relative distance between nodes indicates degrees of similarity. Node color indicates significance, and node size indicates the background size of the biological process (see legend). Representative processes are labeled. Black text denotes processes that were significantly altered (limited to proteins with ≥2-fold change). Blue text denotes significantly altered processes when all significant data is included, irrespective of fold change.

As a first step to characterizing cell signaling in CF neutrophils, we examined their proteome. Peripheral blood neutrophils were collected from 10 non-CF control subjects and 14 pwCF by negative selection and protein lysate was generated. Protein from 3 CF donors were removed because of poor sample quality and several proteins were judged to be contaminants that were excluded from the analysis (supplemental table 1). All remaining samples were analyzed by LC-MS/MS. Of the 1929 proteins identified, 402 were significantly upregulated in lysate from CF neutrophils and 1052 were significantly downregulated (Fig. 1A, Supplemental Data File). A summary of the proteins with the greatest fold change is shown in Table 2 and all data are shown in the supplemental data file. Interestingly, proteins involved in cell signaling, including several kinases, were downregulated (e.g. NADK, MAP2K2). Consistent with plasma membrane ion channels being very hydrophobic, we did not detect Orai1, CFTR or other ion channels of interest. However, other proteins involved in Ca^2+^ signaling showed small but significant downregulation (e.g. STIM1, PLC2B, PLCG2 and the plasma membrane Ca^2+^ pump ATP2B4; Supplemental Data File). Inositol triphosphate (IP_3_) formation is required for activation of SOCE, and several proteins involved in IP_3_ metabolism were also altered in pwCF-derived neutrophils (Supplemental Data File). From an innate defense perspective, neutrophil elastase was not significantly altered, while SPLI was moderately upregulated. Matrix metalloproteinase-8 (MMP8), MMP9, cathepsin D, G and S displayed small but significant amounts of upregulation, while other proteases (eg. cathepsin B, serine protease 1 (PRSS1) and caspase 14) were downregulated. In contrast, myeloperoxidase was significantly upregulated (supplemental data file).

To investigate the most affected proteins in more detail, we selected those with a ≥2-fold change in mean relative abundance and Q-value ≤0.1. Hierarchical clustering (one minus Pearson method) differentiated non-CF from CF samples (Fig. 1B). 9/11 CF donors were taking CFTR corrector/modulator treatment at the time of donation and hierarchical clustering did not differentiate CF neutrophils from patients using or not using ETI. Gene ontology (GO) identified significant associations between the altered proteins and 8 cellular compartments: the extracellular region, extracellular space, extracellular exome, vesicles, tertiary granules, secretory granules, specific granules, and blood microparticles (Fig. 1C). Further, the analysis software indicated that the proteins shared significantly more functional relationships than expected for a random group of proteins this size (*p* = 1.49 × 10^−6^; Fig. 1C). Neutrophils are highly specialized immune cells, and this analysis provides further validation that related groups of proteins are collectively up- or down-regulated in CF neutrophils. Significant associations were found for GO biological processes associated with exocytosis/degranulation, cell activation/immune response and platelet derived growth factor signaling (Fig. 1D). When all significantly altered proteins were included, significant associations for GO biological processes including metabolic/catabolic and regulatory processes were also detected (Fig. 1D). We identified several ion channels, transporters, and regulatory pathways with altered expression (Table 2). Proteins involved in ROS metabolism and pH regulation were also dysregulated (Table 2).

### 3.2. CF neutrophils display dysregulated SOCE that inversely correlates with FEV1

**Fig. 2.**
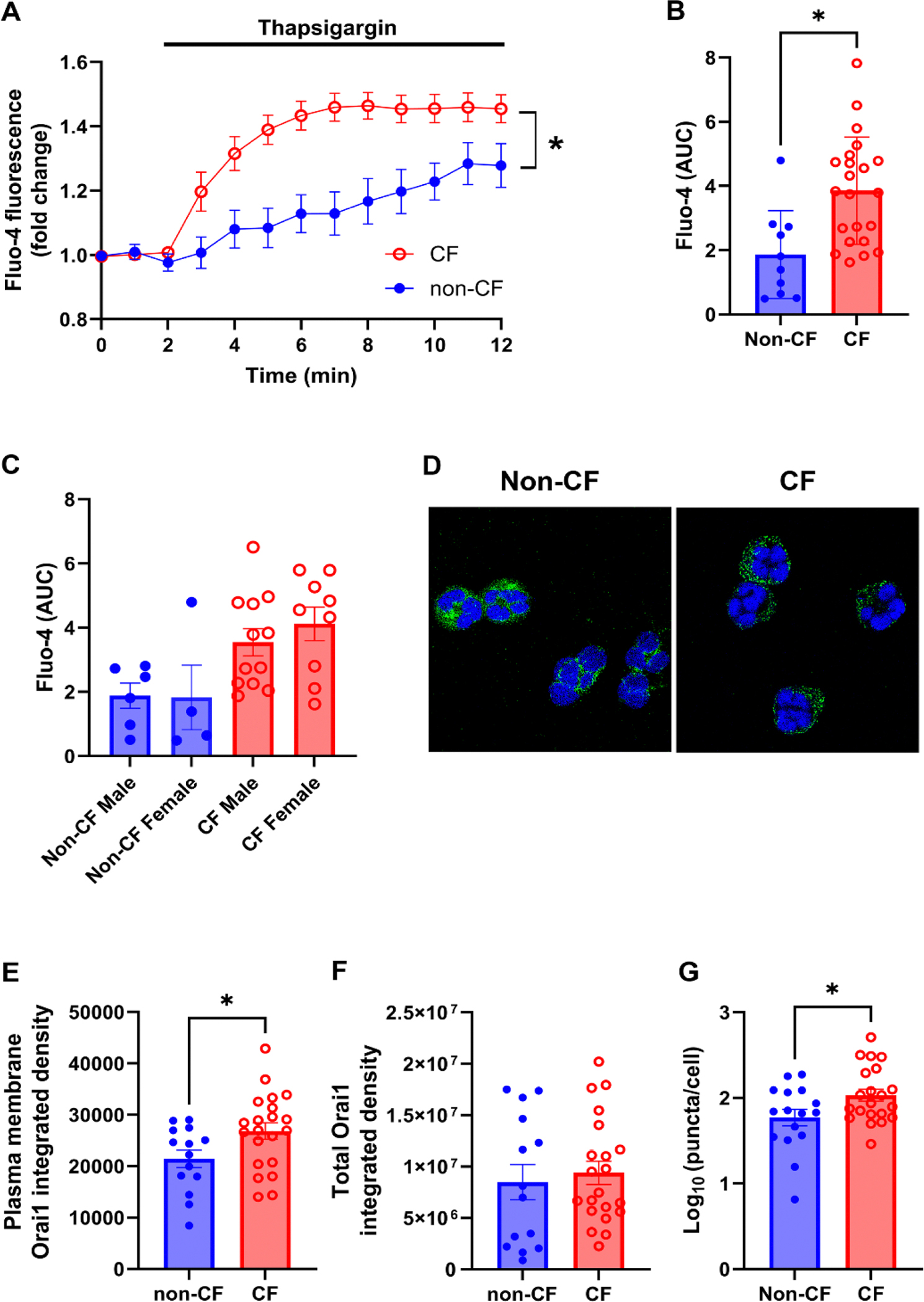
SOCE is upregulated in CF patient neutrophils. (A) Mean traces of fluo-4 fluorescence as a marker of cytoplasmic Ca^2+^ in non-CF and CF neutrophils before and after stimulation with 1 μM thapsigargin. (B) Summary data showing mean area under the curve (AUC) for non-CF and CF neutrophilic fluo-4 responses. (C) AUC summary fluo-4 data separated by sex. (D) Representative confocal microscopy images of non-CF and CF neutrophils stained with antibodies against Orai1 (green) and DAPI (DNA, blue). (E) Orai1 integrated density in the plasma membrane. (F) Total Orai1 integrated density. (G) Orai1 puncta count per neutrophil. Data are mean ± S.E.M. Imaging data is from 3 images per donor. All other data is n = 10 non-CF and 21 CF donors. * = p < 0.05 different to non-CF donors.

Since SOCE plays a major role in regulating neutrophil maturation and activation [26], we next evaluated SOCE in more detail. Peripheral blood neutrophils were isolated from the blood of non-CF and CF donors (See Table 1 for demographics). The majority of CF donors were taking ETI at the time of donation with the remainder not on modulator therapy. SOCE was stimulated using the SERCA pump inhibitor, thapsigargin, and cytoplasmic Ca^2+^ levels were measured using the Ca^2+^-sensitive dye, fluo-4. CF neutrophils exposed to 1 μM thapsigargin displayed significantly greater increases in cytoplasmic Ca^2+^ than non-CF controls, suggesting impaired Ca^2+^ homeostasis (Fig. 2A, B). Interestingly, in addition to the magnitude of the response being different between genotypes, the rate of activation (slope) was also significantly different (non-CF, 0.033 ± 0.012; CF, 0.085 ± 0.009; p<0.001). We have previously shown that both SOCE, and Ca^2+^ dependent responses are estrogen sensitive in pwCF [27]. However, in this current study, no sex effects were observed (Fig. 2C). Similarly, there were no significant differences in Ca^2+^ signaling ± ETI (supplemental figure 1A, B) or for pwCF who cultured positive vs negative for either *P. aeruginosa* or *S. aureus* (supplemental figure 2). Next, we immunostained Orai1 and imaged by confocal microscopy. To differentiate between Orai1 in the plasma membrane and internalized Orai1, we drew a 750 nm region of interest around the plasma membrane, vs a second intracellular region of interest (see supplemental methods). Immunostaining for Orai1 revealed an increase in plasma membrane Orai1 staining in CF neutrophils (Fig. 2D, E) but not an increase in total Orai1 expression (Fig. 2D, F). Orai1 oligomerization into puncta formation is requisite for Orai1 opening and subsequent Ca^2+^ influx. Indeed, in CF lungs, Orai1 is more likely to form puncta than in non-CF lungs [18]. We also found that CF neutrophils displayed increased Orai1 puncta formation compared to non-CF neutrophils, suggesting that Orai1 remained more active (Fig. 2G).

### 3.3. ELD607 inhibits Orai1 in non-CF and CF neutrophils

**Fig. 3.**
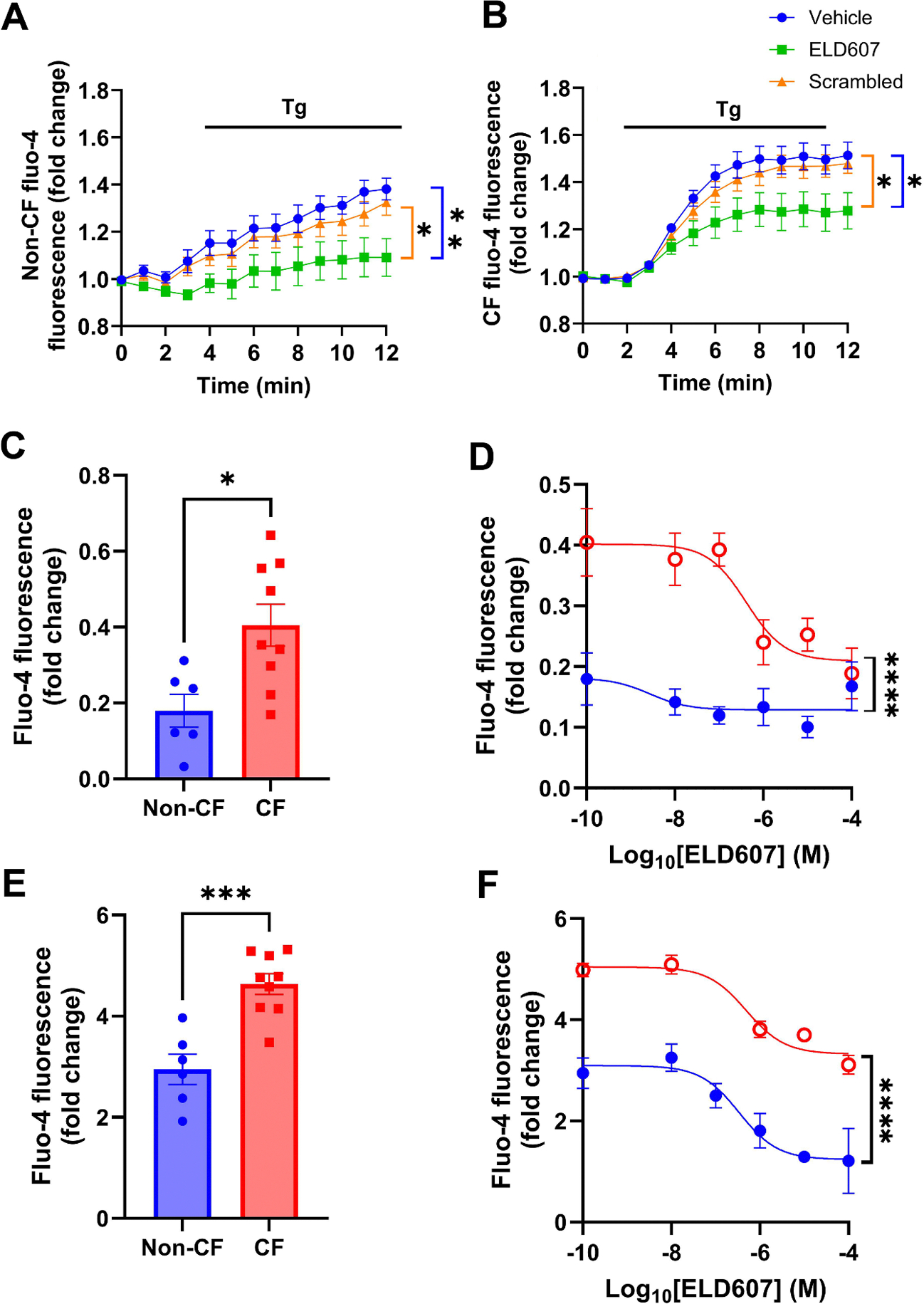
ELD607 inhibits SOCE in non-CF and CF neutrophils. (A) and (B) Mean changes in fluo-4 fluorescence as a marker of cytoplasmic Ca^2+^ levels in non-CF and CF neutrophils respectively before and after stimulation with 1 μM thapsigargin (Tg). Before stimulation, neutrophils were exposed to vehicle (0.1% DMSO in PBS), 10 μM ELD607 or scrambled peptide (control) for 3 h. N = 10 non-CF and 21 CF donors. Stats show ELD607 vs vehicle or scrambled peptide based on color. (C-F) Isolation of ER Ca^2+^ release vs Ca^2+^ influx across the plasma membrane in non-CF and CF Neutrophils. Neutrophils were stimulated with thapsigargin (1 μM) in the absence of extracellular Ca^2+^ to allow the release of Ca^2+^ stores to be measured. Extracellular Ca^2+^ was then returned so that extracellular Ca^2+^ influx could be measured. (C) Mean fold change in Fluo-4 fluorescence during ER store release in non-CF and CF neutrophils. (D) Dose responses for ER Ca^2+^ release vs ELD607 exposure (3 h pre-treatment) in non-CF and CF neutrophils. (E) Mean fold change in fluo-4 fluorescence during Ca^2+^ influx in non-CF and CF neutrophils. (F) Dose responses for Ca^2+^ influx vs ELD607 exposure (3 h pre-treatment) in non-CF and CF neutrophils. Data from 2 non-CF and 3 CF donors performed in triplicate. * = p < 0.05, ** = p < 0.01, *** = p < 0.001, **** = p < 0.0001, different to non-CF donors.

We have previously shown that ELD607 reduces neutrophilia and neutrophil elastase release in both *Pseudomonas aeruginosa*- and *Staphylococcus aureus-*infected mice [21]. However, in this previous study, we did not directly test the effects of ELD607 on neutrophils. We hypothesized that inhibiting SOCE in CF neutrophils may reduce the secretion of neutrophil elastase and other proteins associated with CF lung disease. To validate ELD607’s specificity for Orai1 over Orai2 and Orai3, we first evaluated ELD607 function in HEK293T cells that had Orai1, Orai2 and Orai3 stably knocked out [28]. In these triple knockout cells, ELD607 had no effect on SOCE (supplemental figure 3). Similarly, after transfection of either Orai2 or Orai3, these cells remained relatively insensitive to ELD607. In contrast, transfection of Orai1 restored the sensitivity to ELD607 seen in wild-type HEK293T cells (supplemental figure 3), indicating that ELD607 is specific for Orai1 over Orai2 and Orai3. Next we evaluated ELD607’s ability to inhibit SOCE in non-CF and CF neutrophils. Consistent with previous studies [12] and Fig. 2, global Ca^2+^ signaling was upregulated in CF neutrophils relative to non-CF neutrophils (Fig. 3A, B). As an additional control, we used a scrambled version of ELD607 (negative control peptide) which had no effect on SOCE in either group (Fig. 3A–B). ELD607 significantly inhibited SOCE regardless of genotype (Fig. 3A–B). In absolute terms, ELD607 had a greater effect on Ca^2+^ signaling in non-CF neutrophils. However, the relative amount inhibition was similar in both groups.

SOCE has 2 phases: release of Ca^2+^ from ER stores and Ca^2+^ influx from the extracellular space via Ca^2+^ channels such as Orai1. To characterize these phases in non-CF and CF neutrophils, extracellular Ca^2+^ was removed by washing cells with Ca^2+^-free Ringer’s solution immediately before experimentation. Ca^2+^ stores were depleted with the SERCA pump inhibitor thapsigargin. Once the stores had emptied, extracellular Ca^2+^ was reintroduced and Ca^2+^ influx was measured (Fig. 3C–F). A small amount of ER store depletion was detected in both groups, and CF neutrophils displayed a small but significant increase in ER store Ca^2+^ release relative to non-CF neutrophils (Fig. 3C). ELD607 had little effect on ER Ca^2+^ release in non-CF neutrophils, but induced a dose-dependent inhibition of ER Ca^2+^ release in CF neutrophils (IC_50_ = 4.13 × 10^−7^ M; Fig. 3D). These curves were significantly different (p<0.0001), perhaps reflecting a small increase in ER size in CF neutrophils, as has previously been described for CF airway epithelial cultures [29]. In contrast, Ca^2+^ influx across the plasma membrane was ~10-fold greater than ER Ca^2+^ release regardless of genotype, and was significantly greater in CF relative to non-CF neutrophils (Fig. 3E). ELD607 treatment dose-dependently inhibited Ca^2+^ influx in both non-CF and CF neutrophils (non-CF IC_50_ = 3.40 × 10^−7^ M; CF IC_50_ = 4.93 × 10^−7^ M; p<0.0001; Fig. 3F).

**Fig. 4.**
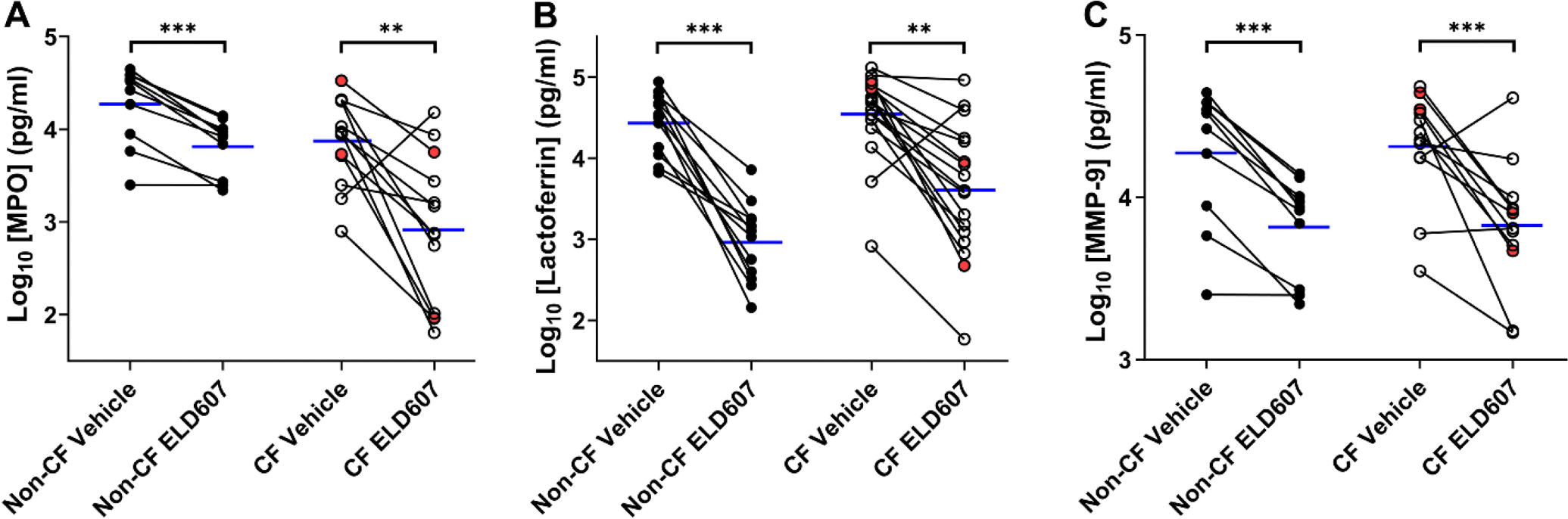
ELD607 inhibits degranulation in non-CF and CF neutrophils. Neutrophil degranulation (A-C) was assayed by measuring the concentration of representative constituents of (A) primary granules, MPO (non-CF, n = 9; CF, n = 13); (B) secondary granules, lactoferrin (non-CF, n = 12; CF, n = 17) and (C) tertiary granules, MMP-9 (non-CF, n = 9; CF, n = 12). Non-CF and CF neutrophils were pretreated with vehicle or ELD607 (10 μM, 3 h) and degranulation was stimulated with 1 μM thapsigargin for 30 min. ** = p< 0.01, *** = p< 0.001 different as indicated using paired t-tests. Blue lines = mean values. 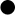, non-CF; ○ CF taking ETI; 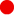, CF ineligible for/not taking ETI.

Multiple neutrophil functions, including neutrophil degranulation, are Ca^2+^ dependent [16]. Neutrophil degranulation results in an increase in several proteases in the lung, including neutrophil elastase, a key driver of CF lung damage [30]. Accordingly, we investigated the impact of ELD607 on neutrophil degranulation as a marker of Ca^2+^-dependent neutrophilic function. We used a multiplex assay to quantify key granule proteins released into media after a 3 h pretreatment with 10 μM ELD607 or vehicle control followed by a 30 min stimulation with 1 μM thapsigargin. Levels of myeloperoxidase (MPO; primary granules), lactoferrin (specific granules) and matrix metalloproteinase-9 (MMP9; tertiary granules) release were similar between non-CF and CF neutrophils (Fig. 4A–C). ELD607 treatment significantly reduced markers of all granules in both non-CF and CF neutrophils, including two pwCF who were not taking CFTR modulators, suggesting that it can exert an anti-inflammatory effect.

**Fig. 5.**
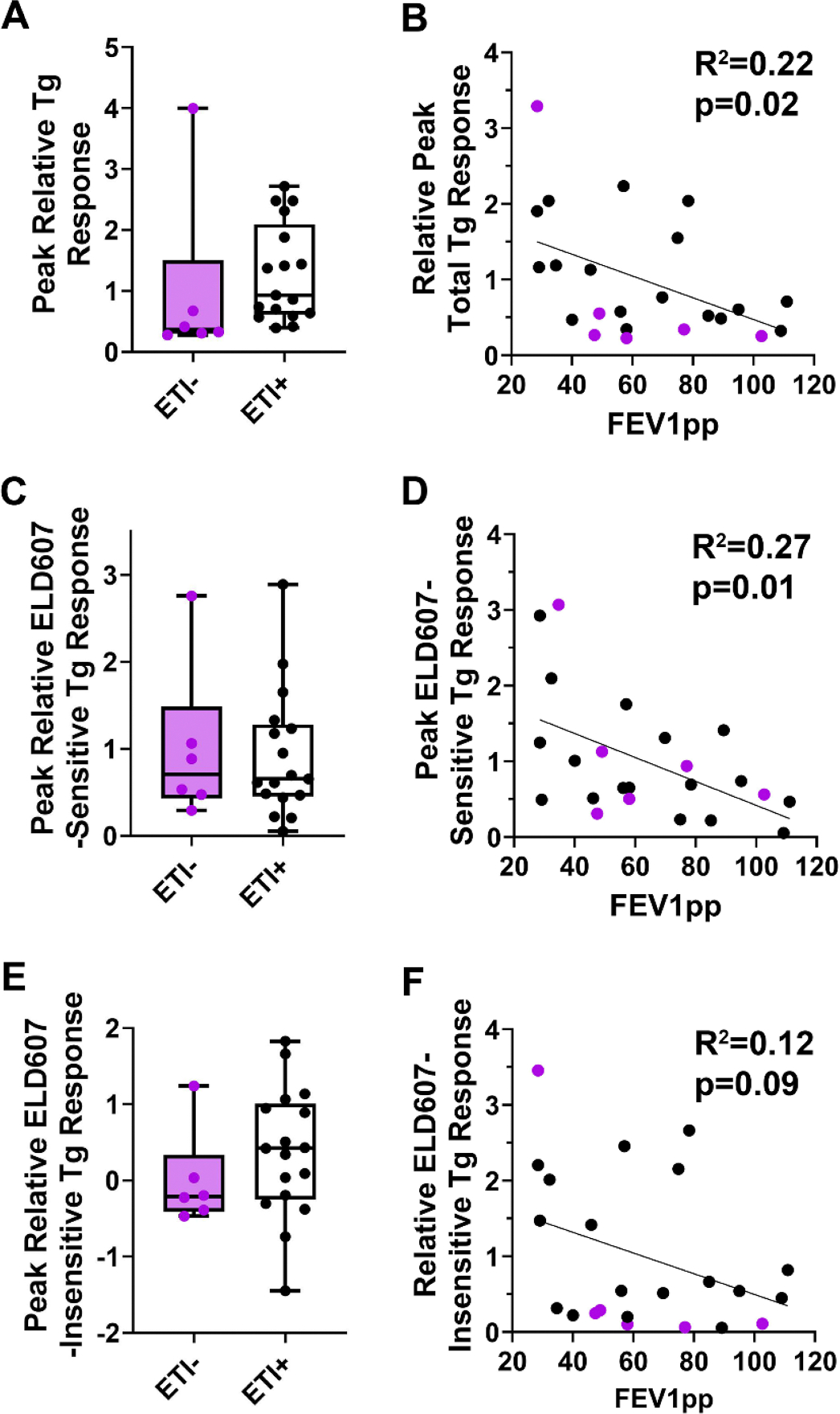
SOCE inversely correlates with FEV1 in CF patients and is independent of ETI usage. (A) Peak total thapsigargin (Tg)-induced Ca^2+^ response in neutrophils from pwCF not taking (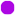) or taking (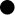) ETI. (B) Graph showing thapsigargin stimulated Ca^2+^ response vs FEV1pp. 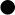 indicate pwCF not using ETI. Black line indicates linear regression. (C) Peak thapsigargin-induced Ca^2+^ response that was inhibited by ELD607 in neutrophils from pwCF not taking (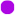) or taking (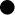) ETI. (D) Graph showing ELD607-sensitive Ca^2+^ response vs FEV1pp. 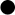 indicate pwCF not using ETI. Black line indicates linear regression. (E) Peak residual thapsigargin-induced Ca^2+^ response that was insensitive to ELD607 in neutrophils from pwCF not taking (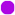) or taking (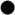) ETI. (F) Graph showing ELD607-insensitive Ca^2+^ response vs FEV1pp. 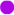 indicate pwCF not using ETI. Black line indicates linear regression. Each data point is an individual subject.

Finally, we compared the impact of ETI on SOCE in CF-derived neutrophils, and SOCE to CF lung function. Peak thapsigargin-induced Ca^2+^ entry was not significantly different between CF neutrophils from subjects taking or not taking ETI (Fig. 5A). Interestingly, peak thapsigargin-induced Ca^2+^ entry showed a significant inverse correlation with lung function (FEV1pp; Fig. 5B). We next evaluated the ELD607-sensitive fraction of SOCE. The (Orai1-mediated) ELD607-sensitive fraction of SOCE was also not different ± ETI (Fig. 5C). Similarly, this portion of Ca^2+^ influx also inversely correlated against FEV1pp (Fig. 5D) and the correlation coefficient and significance increased slightly compared to Fig. 5B. We then compared the ELD607-insensitive fraction of SOCE, which was also unaffected by ETI (Fig. 5E). Interestingly, this fraction did not significantly correlate with lung function (Fig. 5F).

## 4. Discussion

Increased SOCE has previously been described in CF neutrophils and may be causal for CF neutrophil hyperactivity [12]. However, Orai1’s involvement in SOCE in neutrophils has not been extensively studied, and the impact of CFTR modulator therapies on Orai1/SOCE is not well-understood. To globally assess the impact of ETI on CF neutrophils, we first performed a proteomic analysis. This analysis identified multiple proteins that were differentially expressed in CF-derived neutrophils relative to neutrophils from non-CF participants, even though the majority of pwCF in this part of the study had used ETI for >1 year (Fig. 1, supplemental figure 1B). These data indicated that CF neutrophils are not normalized by ETI. Interestingly, beyond the proteins listed in supplemental data file 1, several SOCE-related proteins were differentially expressed in CF neutrophils (Table 1). For example, there was a ~2.5-fold increase in GPR84 in CF neutrophils. Activation of GPR84 initiates Ca^2+^ mobilization and is typically proinflammatory [31]. GPR84 activation is typically upregulated following exposure to LPS in mice [32], suggesting that increased GPR84 may be a response to inflammation rather than a direct effect of CFTR dysfunction. Inositol triphosphate (IP_3_) is a key second messenger that is formed following activation of Gq-linked GPCRs that can initiate SOCE by mobilizing ER Ca^2+^ release [33]. Interestingly, proteins associated with inositol metabolism were significantly downregulated including inositol monophosphatase 2 (IMAP2). IMAP2 inhibition by lithium leads to a cellular accumulation of inositol phosphate, a precursor of IP_3_, the key molecule that activates SOCE. Thus, CF neutrophils may be predisposed to a greater activation of SOCE. Enzymes that modulate the inositol pathway have previously been shown to be affected in CF tissues [34]. However, more experiments will be needed to confirm or refute whether downregulation of IMPA2 leads to greater IP_3_ formation. In addition to changes in potential IP_3_ metabolism, Calcium Release Activated Channel Regulator 2A (CRACR2A) was also downregulated in CF neutrophils (Table 1). CRACR2A is a cytoplasmic Ca^2+^-sensing protein that stabilizes STIM1 and Orai1 at ER/plasma membrane junctions [35]. Again, more experiments will be required to understand the effect of downregulated CRACR2A on CF SOCE, but this may account in part for the altered activation kinetic seen in CF vs non-CF neutrophils (Fig. 2A).

MPO is a major peroxidase secreted from neutrophils which contributes to the formation of hypochlorous acid. Both exaggerated and diminished ROS production in CF neutrophils have been reported [36]. ELD607 reduces MPO production similarly in both non-CF and CF neutrophils, suggesting that it may be able to influence ROS production (Fig. 4). Other proteins involved in ROS and pH homeostasis were also altered in CF neutrophils (Table 1). Peroxiredoxins are thiol peroxidases involved in antioxidant defense and redox signaling. They are hypothesized to be important in neutrophils for handling the large amount of oxidants produced by these cells [37]. Peroxiredoxin-2 was upregulated up ~4.5-fold while peroxiredoxin-6 was downregulated nearly 2-fold in CF neutrophils. During neutrophil activation, peroxiredoxin-6 translocates to the plasma membrane and supports NADPH oxidase activity [38]. Thus, reduced peroxiredoxin-6 expression may contribute to impaired NADPH oxidase activity in CF neutrophils [12]. Collectively, the dysregulation of multiple key processes indicated that CFTR modulators are insufficient to normalize CF neutrophil protein expression. Interestingly, systemic inflammation (neutrophil number and circulating cytokines) has been shown to be significantly reduced in pwCF taking ETI [9,39] and the impact of persistently abnormal neutrophils in CF patients taking ETI remains to be determined.

Since SOCE plays a major role in regulating neutrophil activation and degranulation [40], we performed follow up studies where we directly assessed SOCE in non-CF vs pwCF-derived neutrophils. We found that CF neutrophils displayed significantly greater increases in cytoplasmic Ca^2+^ than non-CF controls following thapsigargin stimulation (Figs. 2A&B). ER Ca^2+^ release was only a trivial component of overall SOCE, that was dwarfed by Ca^2+^ influx across the plasma membrane, suggesting that proteins involved in Ca^2+^ influx are the best target to reduce SOCE in CF neutrophils (Fig. 2 D–F). We observed increased plasma membrane Orai1 levels (Fig. 2E) and increased baseline Orai1 activation (increased puncta formation in the absence of agonists; Fig. 2G). These data indicated that the increased SOCE was at least in part mediated by increased Orai1 expression, and is similar to the increased puncta formation that we have previously seen in CF lungs [18].

We have previously reported that Ca^2+^ signaling is influenced by estrogen in pwCF airways [27]. However, we did not observe any gender differences in SOCE in neutrophils (Fig. 2C). Circulating neutrophil levels fluctuate during the menstrual cycle [41] and sex-specific differences in neutrophil function have previously been described [42,43]. It may have been that all our female participants had low circulating estrogen levels at time of sample, or that SOCE in neutrophils is less-estrogen sensitive than SOCE in airway epithelia. Importantly, most CF participants were taking CFTR modulator therapy at the time of blood collection, and we did not observe significant differences in SOCE between pwCF taking or not taking ETI (supplemental figures 1A, B). To the best of our knowledge, CFTR stability has not been measured in neutrophils, but it typically ranges from several hours for CFTR^ΔF508^ to days for wild-type CFTR [44]. Thus, in the ~2–3 h between neutrophil isolation and measurement of SOCE, it is unlikely that we were experiencing significant CFTR washout. Our ex-patient data contrasted with the *in vitro* studies of Robledo-Avila *et al.,* who found that CFTR potentiation with VX770/Kalydeco was sufficient to normalize SOCE and CF neutrophil bacterial killing [12]. This disparity may reflect differences in the bioavailability of modulators *in vivo* vs *in vitro.* Further, our data may indicate that the rescue of CFTR function by ETI is insufficient to normalize CF neutrophils. Alternatively, the persistence of elevated SOCE in freshly-isolated CF neutrophils may have been a response to ongoing systemic inflammation [9], or, there may be a lack of plasticity in CF neutrophils. However, a limitation of our study is that we were not able to longitudinally assess pwCF neutrophils pre/post ETI, and it is also possible that ETI reduced CF neutrophilic inflammation, but not into the normal range. However, this is unlikely since we did not detect a difference in SOCE between pwCF taking or not taking ETI (Fig. 5).

The role of SOCE in neutrophil activation is well-established is required for degranulation, activation of NAPH-Oxidase and subsequent generation of reactive O_2_ species [40]. Ca^2+^ also alters cytoskeletal dynamics to facilitate neutrophil migration into inflamed tissues [40]. Both mice and human neutrophils express Orai1. However, Orai1’s requirement for neutrophil function is controversial. For example, Orai1 knockdown in the HL-60 neutrophil cell line, and in mice, reduced neutrophil functions [45,46], while another study found that C5a-mediated neutrophil recruitment required Orai1 [47]. However, in one patient with an Orai1 loss-of-function mutation, only modest effects on neutrophil SOCE were observed [48]. Moreover, Ca^2+^ influx in CF neutrophils was recently attributed to increased transient receptor potential (TRP) channel activity [12]. These data were based on inhibition of SOCE by 2-aminoethoxydiphenyl borate (2-ABP), a broad spectrum SOCE antagonist that also inhibits Orai1 and STIM1 [49]. ELD607 is a highly specific Orai1 antagonist that has little effect on Orai2 and Orai3 (extended data Fig. 3). We found that ELD607 markedly attenuated Ca^2+^ influx in non-CF neutrophils (Fig. 3), and in absolute terms restored Ca^2+^ from influx CF neutrophils to non-CF levels. Proportionally, the amount of ELD607 inhibition was similar and Ca^2+^ signaling was not 100% inhibited suggesting that other channels beyond Orai1 are involved in Ca^2+^ signaling in neutrophils. Moreover, additional non-Orai1 Ca^2+^ channels were likely recruited to CF neutrophil plasma membranes to help generate increased SOCE, and further studies will be required to determine which additional channels may be upregulated in CF neutrophils. Additional channels with different activation kinetics may also have explained the difference in activation rates of Ca^2+^ influx (Fig. 2A, B). Of note, based on human genetic studies, it has previously been determined that ≥ 30% residual Ca^2+^ influx is protective against immunosuppression [50]. Thus, given that pwCF are often immunocompromised, not fully inhibiting SOCE is a desirable trait for any future therapy.

ELD607 is a potential therapeutic that reduces neutrophil influx into the lungs of mice following infection with CF-relevant bacterial infections (e.g. *P. aeruginosa, S. aureus* and MRSA) leading to reduced lung injury and increased survival [21]. Interestingly, depletion of alveolar macrophages in these mice abolished the beneficial effects of ELD607. However, these studies were performed on wild-type mice that likely did not exhibit SOCE hyperactivity. Our data indicate that ELD607 may also exert direct effects on CF neutrophils, which may beneficially reduce CF inflammation (Figs. 3,4). In addition to being a potential therapeutic, due to its specificity, ELD607 is a useful tool for probing endogenous Orai1 function in relevant immune cell types. ELD607 dose-dependently inhibited Orai1/SOCE in both non-CF and CF neutrophils (Fig. 3) and significantly inhibited neutrophil degranulation (Fig. 4), suggesting that Orai1 inhibition by ELD607 has functional consequences. For the neutrophil degranulation assay, we were only able to recruit two pwCF who were not taking CFTR modulators, so we cannot make any robust statistical comparisons between pwCF taking and not taking ETI. However, these two subjects were not outliers, and grouped around the means (Fig. 4), suggesting that as with Ca^2+^ signaling, ETI may not have a significant impact on CF neutrophil degranulation. A limitation of our study is that we only explored the SOCE response to thapsigargin. This compound bypasses GPCRs to directly stimulate ER Ca^2+^ release (and subsequent Orai1 activation). However, agonists such as ATP or LPS can stimulate GPCRs to initiate SOCE, and the impact of ELD607 on physiological Ca^2+^ responses should be explored in the future. An additional limitation is that we only focused on degranulation as the Ca^2+^ dependent/ELD607-sensitive endpoint and further investigation into the effects of ELD607 on other relevant endpoints including neutrophil migration, ROS production and antimicrobial activities are also warranted.

SOCE remained upregulated in pwCF compared to healthy non-CF controls. Previous groups have indicated that CFTR directly interacts with Orai1 to modulate SOCE levels [51] and ETI may not provide enough CFTR correction to restore SOCE. Alternatively, persistent infection in pwCF may have provided an ongoing stimulus to perpetuate upregulated SOCE. Further studies, including non-CF patient groups with chronic lung infections will be needed to better understand this phenomenon. Neither *P. aeruginosa* nor *S. aureus* infection status affected SOCE levels (extended data Fig. 2). However, these patients may have been colonized with other organisms which could have influenced our outcomes, and for this study, we did not perform a detailed microbiological characterization of our subjects. In contrast, peak thapsigargin-induced SOCE inversely correlated with lung function (Fig. 5B). For this assay, we performed a global SOCE study, where we measured the integration of ER Ca^2+^ release and Ca^2+^ entry through not only Orai1 but also potentially through other Orai channels or unrelated Ca^2+^ channels (e.g. TRP channels). Interestingly, when we compared the fraction of SOCE that was inhibitable by ELD607 against lung function, the correlation and significance increased, suggesting that Orai1 is a good biomarker of CF lung disease.

CF neutrophils with elevated SOCE undergo more degranulation, leading to greater release of proteases/pro-inflammatory mediators. Therefore, increased SOCE may be a driving force for declining CF lung function. However, there is much to do to fully understand this phenomenon, and our data identify an association, not a causality. Importantly, these data indicated that abnormal cell signaling in CF neutrophils is at least partially related to lung-disease severity in these patients. To date, several blood biomarkers have been identified as predictors of CF lung disease [52]. Most biomarkers (e.g. cytokines) represent only a single facet of inflammation and SOCE is convergent for multiple inflammatory pathways. Peripheral blood neutrophils are relatively easy to obtain and hence, SOCE in CF neutrophils may represent an important and accessible biomarker of lung health. Thus, the relationship between peripheral blood neutrophil Ca^2+^ homeostasis and CF lung disease warrants further investigation. While neutrophilic inflammation is well-described in the lung, neutrophilia occurs at all CF epithelial sites including the gut, liver and pancreas and is a major cause of damage in these organs [36]. Indeed, neutrophil lysis causes neutrophil elastase release that can cause significant damage in these organs. In our previous studies, we found that inhaled ELD607 abolished neutrophilia caused by bacterial infections [53]. Systemically delivered ELD607 may be useful to treat non-pulmonary neutrophilia and prevent progressive multi-organ damage in pwCF. However, further studies will be needed to evaluate ELD607 effectiveness, pharmacokinetics and safety when delivered systemically.

## 5. Conclusions

**Fig. 6.**
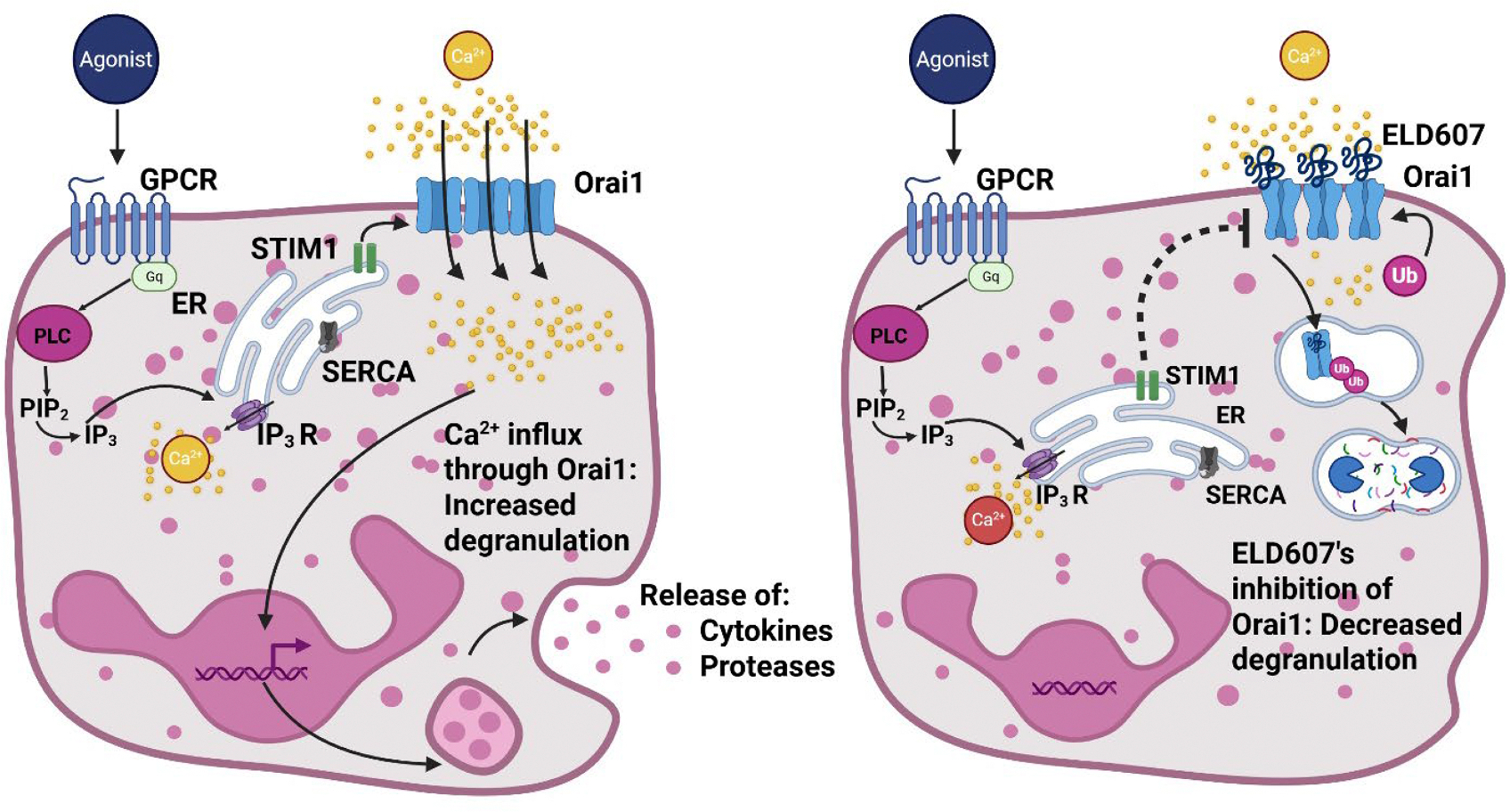
Cartoon of SOCE in neutrophils in the absence (left) and presence (right) of ELD607. Left, activation of G_q_-linked GPCRs by appropriate agonists (e.g. ATP) leads to formation of inositol triphosphate (IP_3_) and ER store Ca^2+^ depletion. This in turn causes STIM1 to rapidly aggregate at ER-plasma membrane junctions where it causes Orai1 to activate and aggregate to provide a second, amplifying wave of Ca^2+^ which triggers neutrophil degranulation, as well as activating other Ca^2+^-dependent processes (not shown). Right, ELD607 binds to Orai1, causing it to be ubiquitinated, internalized and degraded. This limits the amount of Orai1-mediated Ca^2+^ influx, reducing neutrophil degranulation. Created in https://BioRender.com.

Collectively, our proteomic and cellular data indicate that CF neutrophils remain abnormal despite most subjects taking ETI. Since Orai1 was upregulated in CF neutrophils (Figs. 2, 3) and in CF lungs [18], we hypothesize that Orai1 inhibition is an attractive target to reduce CF neutrophilic inflammation. Orai1 inhibition is predicted to have a broad anti-inflammatory effect [16]. STIM1 is directly upstream of Orai1 in the SOCE signaling cascade (Fig. 6). To date, ibuprofen is the only anti-inflammatory therapy approved for CF patients [54] and since inflammation is an ongoing problem in CF patients [55], new therapies are urgently needed. ELD607 will require a significant amount of additional testing before it is ready for clinical trials, but is promising since it can effectively reduce neutrophil degranulation (Fig. 6). In conclusion, these data highlight the potential for neutrophil SOCE responses to be used as a novel biomarker of disease progression. Further, we demonstrated that ELD607 inhibits SOCE in non-CF and CF neutrophils to reduce the release of granule contents. This would be predicted to reduce levels of neutrophil elastase and other proteases in the CF lung. Rebalancing SOCE in CF neutrophils with therapeutics such as ELD607 may be beneficial in the management of CF airway inflammation.

## Supplementary Material

Supplemental Material

Supplementary materials

Supplementary material associated with this article can be found, in the online version, at doi:10.1016/j.jcf.2025.08.014.

## Figures and Tables

**Table 1:** Subject demographics.

Figure	Number of Subjects	Age (years)	FEV1pp	Sex (M/F)	% on Modulators	% with at least one copy of ΔF508

1	11	27 ± 5.5	87.3 ± 24.2	8/6	92.9	85.7
2	21	30.7 ± 12.9	63.5 ± 29.9	11/4	73.3	73.3
3	21	29.8 ± 12.1	69.6 ± 30.0	12/6	77.8	72.2
4	17	27.8 ± 6.9	88.2 ± 22.8	10/7	88.2	82.4
5	23	31.1 ± 11.2	67.0 ± 27.3	15/8	73.9	69.0

Data for pwCF who donated blood are shown in Table 1 as mean ± standard deviation or percentage as appropriate. Mean age for healthy non-CF donors was 33.1 ± 9.1 years (9M/7F total).

**Table 2:** Summary of proteins with greatest fold-change and Q-value in CF neutrophils.

Name	Description	Log2 FC	Q-Value

HSPB1	Heat shock protein beta-1	−1.71273	0.007118
DCD	Dermcidin	−1.69216	0.000303
CDSN	Corneodesmosin	−1.52689	0.007406
FLG2	Filaggrin-2	−1.49212	0.003571
HIP1	Huntingtin-interacting protein 1	−1.48183	2.52E-06
TGM3	Protein-glutamine gamma-glutamyltransferase E	−1.46347	0.009918
IGKV3–15	Immunoglobulin kappa variable 3–15	−1.3822	0.00429
LAMTOR3	Late endosomal/lysosomal adaptor, MAPK and MTOR activator 3	−1.37727	0.000418
PEBP1	Phosphatidylethanolamine-binding protein 1	−1.32947	0.000288
CRACR2A	Calcium Release Activated Channel Regulator 2A	−1.31601	0.00815
DSG1	Desmoglein-1	−1.2591	0.009097
DNPEP	Aspartyl aminopeptidase	−1.22361	4.14E-07
RANBP1	Ran-specific GTPase-activating protein	−1.18489	0.000217
GSTM2	Glutathione S-transferase Mu 2	−1.18055	0.001347
IMPA2	Inositol monophosphatase 2	−1.147	6.15E-06
DSP	Desmoplakin	−1.09796	0.004922
EEF1D	Elongation factor 1-delta	−1.08522	6.38E-06
HRNR	Hornerin	−1.07953	0.006785
PDLIM7	PDZ and LIM domain protein 7	−1.06599	1.02E-09
FHL3	Four and a half LIM domains protein 3	−1.06198	1.71E-06
PIN1	Peptidyl-prolyl cis-trans isomerase NIMA-interacting 1	−1.05606	0.006037
APOD	Apolipoprotein D	−1.05479	0.003108
SCRN1	Secernin-1	−1.05289	4.04E-06
HGS	Hepatocyte growth factor-regulated tyrosine kinase substrate	−1.02132	0.000211
ACTR1A	Alpha-centractin (actin-related protein 1A)	−1.0151	7.74E-06
LGALS3BP	Galectin-3-binding protein	1.021244	0.007408
LILRA6	Leukocyte immunoglobulin-like receptor subfamily A member 6	1.060708	0.000106
LBP	Lipopolysaccharide-binding protein	1.144445	0.003209
HP	Haptoglobin	1.189361	3.74E-06
GPR84	G protein-coupled receptor 84	1.294277	0.00021
CREG1	Cellular Repressor of E1A Stimulated Genes 1	1.306042	0.000131
GALNT1	Polypeptide N-acetylgalactosaminyltransferase 1	1.368939	0.000134
IGKV6–21	Immunoglobulin kappa variable 6–21	1.491607	0.000274
CA2	Carbonic anhydrase 2	1.529759	0.000159
FCGBP	IgG Fc-binding protein	1.633013	0.000396
PRG2	Proteoglycan 2	1.785945	0.000955
CP	Ceruloplasmin	1.859472	9.86E-05
PRDX2	Peroxiredoxin-2	2.231358	1.49E-06
CA1	Carbonic anhydrase 1	2.626865	2.38E-05
PRG3	Proteoglycan 3	2.874707	0.000433
SAA1	Serum amyloid A-1 protein	2.912545	0.00025

Non-CF and CF peripheral blood neutrophils were isolated, lysed and protein was collected. Proteins were identified and quantified by LC-MS/MS. Mean Log_2_ Ratio indicates the change in mean abundance of a protein in CF vs non-CF neutrophils. Q value is the false discovery rate (adjusted p-value) of comparisons of mean protein abundance in CF vs non-CF neutrophils. A full list of significantly altered proteins and raw proteomics data is included in the supplement.
