# Supplementary material for: Neutrophil store-operated Ca2+ entry: A correctable biomarker of cystic fibrosis lung disease progression
Source: J Cyst Fibros. Author manuscript; Available in PMC 2026 Mar 18. (PMC12998938; doi:10.1016/j.jcf.2025.08.014)
Supplement: Supplemental Material [file NIHMS2140695-supplement-Supplemental_Material.docx]

**Supplemental Methods and Data for:**

**Neutrophil Store-Operated Ca^2+^ Entry: A Correctable Biomarker of Cystic Fibrosis Lung Disease Progression**

**Joe A Wrennall^1^, Matthew GS Biggart^2^, Charles D Bengston^3^,**

**M Flori Sassano^2^ and Robert Tarran^2^***

^1^Department of Cell Biology and Physiology, University of North Carolina at Chapel Hill, NC, 27599; ^2^Division of Genetic, Environmental and Inhalational Disease, ^3^Division of Pulmonary, Critical Care and Sleep Medicine, Department of Internal Medicine, Kansas University Medical Center, Kansas City, KS, 66103, USA.

## Supplemental Materials and Methods

## Full Methods

### **Solutions and reagents.** A modified Ringer’s solution was used which contained (in mM): 120 NaCl, 12 NaCHO_3_, 24 HEPES, 1.2 MgCl_2_, 5.2 KCl, 10 glucose, 1.2 CaCl_2_. To measure SOCE, a Ca^2+^-free Ringer’s solution was used which contained (in mM): 120 NaCl, 12 NaCHO_3_, 24 HEPES, 1.2 MgCl_2_, 5.2 KCl, 10 glucose, 0.25 EGTA. Chemicals were obtained from Sigma-Aldrich unless otherwise indicated.

### **Peptides.** ELD607 [VHDIVNMLIRG] and scrambled ELD607 [VIRGINDMHVL] were made by Fmoc solid phase synthesis either at UNC-Chapel Hill, Shanghai Royobiotech or AmbioPharm. Powdered peptides were stored at -80°C until required.

**Neutrophil isolation.** Deidentified samples were provided by the University of North Carolina’s Cystic Fibrosis Clinical Translation Core and Environmental Medicine Asthma and Lung Biology biorepository or by the Department of Internal Medicine at KUMC. 5-ml blood samples were collected in EDTA-containing vacuum tubes, placed ice and neutrophils were immediately isolated by negative selection using an EasySep human neutrophil isolation kit (Stem Cell Technologies). Neutrophils were resuspended in RPMI 1640 media supplemented with 10 mM HEPES at 500,000 cells/ml for experimentation. Neutrophils were isolated to be >99% pure. Demographic information is presented in supplemental tables 2 and 3. No difference in SOCE was detected in pwCF neutrophils collected from UNC vs KUMC (supplemental figure 4).

**Proteomics****.** Protein lysate from isolated blood neutrophils was subjected to liquid chromatography with tandem mass spectrometry (LC-MS/MS). 65 µg of each sample were reduced with DTT, alkylated with iodoacetamide, and digested with LysC, and then trypsin overnight. Peptide samples were cleaned using Pierce desalting spin columns, then peptide quantitation was conducted using the Fluorometric peptide BCA assay (Pierce). A pooled sample was created by combining an equal volume of each sample. Each sample was analyzed via LC-MS/MS using a Thermo Easy nLC 1200 – Exploris480 using Data Independent Acquisition, and in a randomized order. The pooled sample was analyzed three times intermittently during the sequence to assess technical reproducibility for a total of 3 pooled sample runs.

**Proteomics Data Analysis**. Proteins were identified and quantified with Spectronaut v17 using a Uniprot Human database (~20,000 proteins). Single hits excluded (only one peptide was identified for the protein). Further data analysis (t-tests, visualization) were also performed in Spectronaut. Data were normalized, and outliers were identified and removed. Significantly altered proteins were screened for contaminants (Supplemental Table 1) and analyzed for functional relationships using DBString [18]. In this analysis, significant interactions (adjusted P-value < 0.05) with gene ontology (GO) terms based on biological process and cellular compartment were assessed. Significant biological processes were mapped as a semantic space using Revigo [19] (www.revigo.irb.hr).

**Fluo-4 Ca^2+^ assays.** Isolated neutrophils were incubated with peptide or vehicle control for 3 h. For the final hour of treatment, cells were loaded with Fluo-4 direct (Thermofisher) for 45 min at 37°C and 15 min at room temperature. For global Ca^2+^ measurements, baseline fluorescent readings were made for 2 min (1 read every 30 s) using a Tecan Infinite M1000 plate reader (ex 494 em 516 nm, bandwidth 5 nm) before thapsigargin (Sigma-Aldrich) was added and the change in emission was recorded for 10 min (1 read every 30 s). To isolate phases of store operated Ca^2+^ entry, cells underwent 3 rounds of centrifugation and resuspension in Ca^2+^-free Ringer’s solution to remove extracellular Ca^2+^. Baseline fluo-4 fluorescence was read for 2 min before cell were stimulated with thapsigargin (Tocris). Reads continued for ~5 min before 1.2 mM Ca^2+^ was added to the extracellular solution and SOCE was measured for ~10 mins. For analysis, all fluo-4 fluorescence reads are normalized to baseline.

**Immunocytochemistry.** Isolated neutrophils were treated for 3 h with vehicle, 10 µM ELD607 or peptide control (scrambled) and were fixed in 4% PFA, washed in PBS, permeabilized in 0.1% Triton X-100 in PBS and blocked overnight (0.1% Triton X-100, 10% FBS, 5% NGS in PBS). Cells were incubated in 1:500 rabbit anti-Orai1 primary antibody (Sigma, SAB3500412) overnight at 4^o^C, washed 3 times in PBS and stained for 3 h at 4^o^C in 1:2000 goat anti-rabbit IgG Dylight-633 secondary antibody (ThermoScientific, 35562). Cells were mounted in VECTASHIELD PLUS mounting media with DAPI and imaged on a Leica SP8 confocal microscope (Dylight 633: excitation 594 nm, emission 600-764 nm; DAPI excitation 405 nm, emission 410-480 nm). Orai1 fluorescence was measured using imageJ (NIH). Neutrophil perimeters were manually defined. The plasma membrane was defined as a 750 nm wide line surrounding the perimeter of the cell. Integrated density was measured for the full area of the neutrophil (total) and in the plasma membrane region. Several neutrophils were measured per image and the mean value was recorded. 3 images analyzed per donor. “Puncta” were defined as spatially localized bright points of fluorescence. They were counted using a strict intensity threshold that only included the brightest pixels of Orai1 fluorescence. Mean puncta count from several cells per imaged was recorded. 3 images were analyzed per donor.

### **Multiplex assay.** Neutrophils were isolated and suspended in RPMI1640 media supplemented with 10mM HEPES at a density of 5x10^5^ neutrophils/ml) Neutrophils were treated with vehicle, 10 µM ELD607 or scrambled ELD607 (control) for 3 h. for the last 30 min of treatment, 1 µM thapsigargin was added to stimulate degranulation. Supernatant was collected and analyzed for a panel of neutrophil granule proteins (MPO, lactoferrin, MMP9) using a Bio-Plex human kit (BioRad) per the manufacturer’s protocol. Plates were then analyzed using a Bio-Plex MAGPIX System (BioRad).

### **Quantification and statistical analysis.** Heat maps were rendered using Morpheus (https://software.broadinstitute.org/morpheus). All other statistical analysis was performed using Graph Pad Prism 9.0. Data were analyzed using Student’s t-test or ANOVA followed by Sidak’s post-test. Paired data were analyzed using a mixed effects model with Holm-Sidak post-test. Doses response curves were compared using the extra sum-of-squares f test.

| **Protein name** | **Protein description** | **# Unique Peptides** | **AVG Log2 Ratio** | **Pvalue** | **Qvalue** | **CF mean** | **Non-CF mean** |
| --- | --- | --- | --- | --- | --- | --- | --- |
| HBB_HUMAN | Hemoglobin subunit beta | 20 | 5.002 | <0.001 | <0.001 | 1686191.875 | 52602.684 |
| HBA_HUMAN | Hemoglobin subunit alpha | 16 | 4.832 | <0.001 | <0.001 | 723802.188 | 25409.025 |
| HBD_HUMAN | Hemoglobin subunit delta | 8 | 3.779 | <0.001 | <0.001 | 76018.023 | 5538.223 |
| HBG1_HUMAN | Hemoglobin subunit gamma-1 | 10 | 3.094 | <0.001 | <0.001 | 1407448.250 | 164885.688 |
| SAA1_HUMAN | Serum amyloid A-1 protein | 3 | 2.913 | <0.001 | <0.001 | 7847.462 | 1042.235 |
| K2C71_HUMAN | Keratin, type II cytoskeletal 71 | 13 | -0.625 | 0.100 | 0.044 | 39091.910 | 60275.496 |
| K1C9_HUMAN | Keratin, type I cytoskeletal 9 | 33 | -0.859 | 0.038 | 0.020 | 218509.609 | 396319.594 |
| K1C27_HUMAN | Keratin, type I cytoskeletal 27 | 8 | -0.875 | 0.023 | 0.014 | 59973.148 | 109987.570 |
| w | Keratin, type II cytoskeletal 1 | 44 | -0.905 | 0.017 | 0.010 | 146778.016 | 274869.219 |
| K2C6A_HUMAN | Keratin, type II cytoskeletal 6A | 2 | -0.994 | 0.017 | 0.011 | 4519.607 | 8999.121 |
| K2C3_HUMAN | Keratin, type II cytoskeletal 3 | 5 | -1.065 | 0.036 | 0.019 | 9385.385 | 19630.422 |
| K22E_HUMAN | Keratin, type II cytoskeletal 2 epidermal | 43 | -1.065 | 0.001 | 0.001 | 46819.922 | 97932.711 |
| K2C5_HUMAN | Keratin, type II cytoskeletal 5 | 22 | -1.234 | 0.001 | 0.001 | 11432.739 | 26893.303 |
| K1C10_HUMAN | Keratin, type I cytoskeletal 10 | 30 | -1.313 | <0.001 | <0.001 | 65220.203 | 162049.828 |
| K2C6C_HUMAN | Keratin, type II cytoskeletal 6C | 47 | -1.461 | 0.002 | 0.002 | 74771.516 | 205859.609 |
| K1C13_HUMAN | Keratin, type I cytoskeletal 13 | 11 | -1.515 | <0.001 | <0.001 | 9001.312 | 25731.299 |
| CDSN_HUMAN | Corneodesmosin | 3 | -1.527 | 0.011 | 0.007 | 3360.615 | 9684.059 |
| K2C6B_HUMAN | Keratin, type II cytoskeletal 6B | 4 | -1.705 | 0.004 | 0.003 | 42349.699 | 138050.672 |
| K1C14_HUMAN | Keratin, type I cytoskeletal 14 | 36 | -1.730 | 0.001 | 0.002 | 86480.648 | 286926.156 |
| K1C17_HUMAN | Keratin, type I cytoskeletal 17 | 13 | -1.749 | 0.016 | 0.010 | 3236.178 | 10875.927 |
| XP32_HUMAN | Skin-specific protein 32 | 2 | -2.201 | 0.059 | 0.029 | 321.171 | 1476.485 |
| KRT83_HUMAN | Keratin, type II cuticular Hb3 | 9 | -2.936 | 0.016 | 0.010 | 1264.178 | 9673.704 |
| KRT84_HUMAN | Keratin, type II cuticular Hb4 | 37 | -3.244 | 0.006 | 0.005 | 3995.031 | 37852.125 |
| KRT86_HUMAN | Keratin, type II cuticular Hb6 | 48 | -3.592 | 0.010 | 0.007 | 86142.008 | 1038814.563 |
| KRT34_HUMAN | Keratin, type I cuticular Ha4 | 17 | -3.597 | 0.030 | 0.017 | 233.402 | 2823.496 |
| KT33B_HUMAN | Keratin, type I cuticular Ha3-II | 20 | -3.648 | 0.049 | 0.025 | 590.300 | 7401.965 |
| KRT85_HUMAN | Keratin, type II cuticular Hb5 | 26 | -3.676 | 0.005 | 0.004 | 2814.037 | 35962.672 |
| K1H1_HUMAN | Keratin, type I cuticular Ha1 | 37 | -4.046 | 0.005 | 0.004 | 50225.336 | 829437.438 |
| KR111_HUMAN | Keratin-associated protein 11-1 | 6 | -7.856 | 0.043 | 0.022 | 5.892 | 1365.408 |

**Supplemental Table 1. Proteins judged to be contaminants that were excluded from the proteomic analysis.** AVG Log2 Ratio indicates the change in mean abundance of a protein in CF vs non-CF neutrophils. Q-value is the p-value of comparisons of mean protein abundance in CF vs non-CF neutrophils that is adjusted for a positive false discovery rate.

| **Donor ID** | **Age** | **Sex** |
| --- | --- | --- |
| 20210308 | 43 | M |
| 20210309 | 50 | M |
| 20210222 | 30 | M |
| 20220127-6 | 24 | F |
| 20220303 | Unknown | Unknown |
| 20220519-1 | 28 | M |
| 20220519-2 | 31 | M |
| 20220630 | 24 | F |
| 20220707 | 24 | F |
| 20220714 | 27 | F |
| 20220726 | 31 | M |
| 20220728 | 41 | F |
| 20220801 | 39 | F |
| 20220804 | 23 | M |
| 20220817-1 | 50 | M |
| 20220817-2 | 28 | M |
| 20220825 | 36 | F |

**Supplemental Table 3.** **Non-CF donor demographics.** All donors were considered healthy and did not have any known pulmonary or systemic disease, current allergies or known viral or bacterial infections.

## Supplemental Figures and Legends

**Supplemental figure 1. Elevated SOCE in CF neutrophils is not rescued by ETI treatment**. Peripheral blood neutrophils were obtained from pwCF and studied immediately. Graph shows peak thapsigargin-stimulated Ca^2+^ responses vs time that ETI was used in days. Each data point represents a separate CF subject.


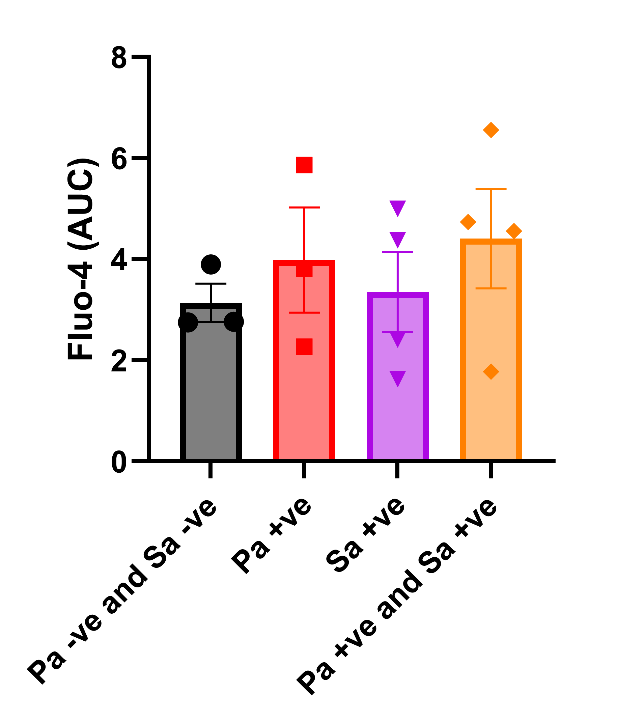


**Supplemental figure 2. Elevated CF neutrophil Ca^2+^ responses are not impacted by *Pseudomonas aeruginosa* (Pa) or *Staphylococcus aureus* (Sa) infection status**. CF blood neutrophil Ca^2+^ responses after thapsigargin stimulation were measured by Fluo-4 fluorescence and grouped based on infection status determined by medical records. Each data point represents a separate CF subject.

**Supplemental figure 3. ELD607 specifically inhibits Orai1.** Dose responses of thapsigargin-induced changes in cytoplasmic Ca^2+^ concentrations. These studies were performed in HEK293T where Orai1, Orai2 and Orai3 had been stably knocked down (triple knockout cells, TKO) or in TKO cells that were transiently transfected with Orai1, Orai2 or Orai3 as indicated. All data are from 7-9 replicates per data point from 3 separate experiments.  Data are shown as mean ± S.E.M. Using the extra sum of squares F-test, the Orai1, 2 and 3 plots were found to be significantly different (p<0.001).


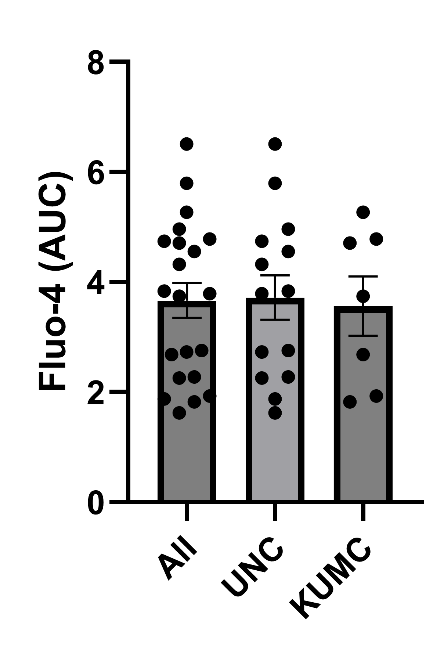


**Supplemental figure 4. Comparison of SOCE in CF neutrophils across both collection sites.** The majority of neutrophils were collected at UNC-Chapel Hill. However, additional neutrophils were collected from 7 pwCF at KUMC. No batch effect was detected.
